# Case report: Idiopathic subglottic stenosis in a girl; successful treatment with macrolides

**DOI:** 10.3389/fped.2022.888282

**Published:** 2022-08-18

**Authors:** Wolfgang Tebbe, Helmut Wittkowski, Johannes Tebbe, Georg Hülskamp

**Affiliations:** ^1^Pädiatrische Pneumologie, Klinik für Kinder - und Jugendmedizin, Clemenshospital, Münster, Germany; ^2^Pädiatrische Rheumatologie und ImmunologieKlinik für Kinder - und Jugendmedizin, Universitätsklinikum Münster (UKM), Münster, Germany; ^3^Allgemeine Pädiatrie, Pädiatrische Pneumologie, Klinik für Kinder- und Jugendmedizin, Universitätsklinikum Münster (UKM), Münster, Germany

**Keywords:** idiopathic subglottic stenosis, child, review, macrolide, therapy

## Abstract

An 8-year-old girl presented with treatment-refractory cough and inspiratory stridor. Bronchoscopies showed progressive scarring leading to narrowing of the proximal trachea (Myer-Cotton Grade 2) and epithelial metaplasia of the tracheal and bronchial mucosa. After excluding other causes of congenital and acquired tracheal stenosis, an idiopathic subglottic tracheal stenosis (iSGS) was diagnosed. Because of the patient's young age, a judicious therapeutic approach seemed appropriate. Therapy with azithromycin, followed by roxithromycin, was started. Symptoms almost completely subsided, spirometry normalized, and endoscopic and histologic findings improved considerably. Therapy has been continued for more than 3 years with normal lung function values, and no compromise on physical activities and development. In instances of iSGS, therapy with macrolides is worth considering before more invasive procedures such as dilatation, laser, intralesional injections, or surgical resection are performed.

## Introduction

Idiopathic subglottic stenosis (iSGS) is a rare progressive disease (1, 2) mainly affecting women around the menopause; reports in children are anecdotal. The etiology remains largely unclear. Treatment options include repeated local injection with corticosteroids, mitomycin, tracheal dilatation, laser coagulation, and surgery with plastic reconstruction or resection of the stenosis with end-to-end anastomosis of the trachea (3–5).

## Case report

An 8-year-old girl presented with a persistent, initially purulent, barking cough over the prior 7 months, that occurred predominantly during the night. Bacterial bronchitis was suspected and a short antibiotic course with erythromycin resulted in slight improvement. Thereafter the non-productive cough worsened again. A cough-variant asthma was suspected. Inhalative steroids and proton-pump-inhibitor treatment (PPI) did not improve symptoms. Codeine had little effect. Clinical examination and routine blood chemistry revealed no other abnormalities. Chest X-ray and spirometry were unremarkable. Bronchoscopy showed a circular deformed, non-stenotic trachea and a granular appearance of the mucosa. BAL and microbial investigations did not show any relevant pathology. Gastroscopy could not demonstrate a gastro-esophageal reflux disease (GERD) but showed a duodenal mucosal atrophy consistent with celiac disease. Serologic markers confirmed the latter. The uncertainty of the cause of the cough led to a therapeutic trial with amoxicillin-clavulanate and omeprazole; inhalative steroids were continued. **9** months after the onset of symptoms, she developed chronic spontaneous urticaria, which was well-controlled by intermittent administration of cetirizine. Coughing persisted.

After 1 year, bronchoscopy noted irregular mesh-like tissue strands in the trachea, starting 3 cm below the glottis over a length of 2–3cm, ending 4 cm above the main carina. Biopsies showed mucosal metaplasia with scattered eosinophils.

**Figure 1:**
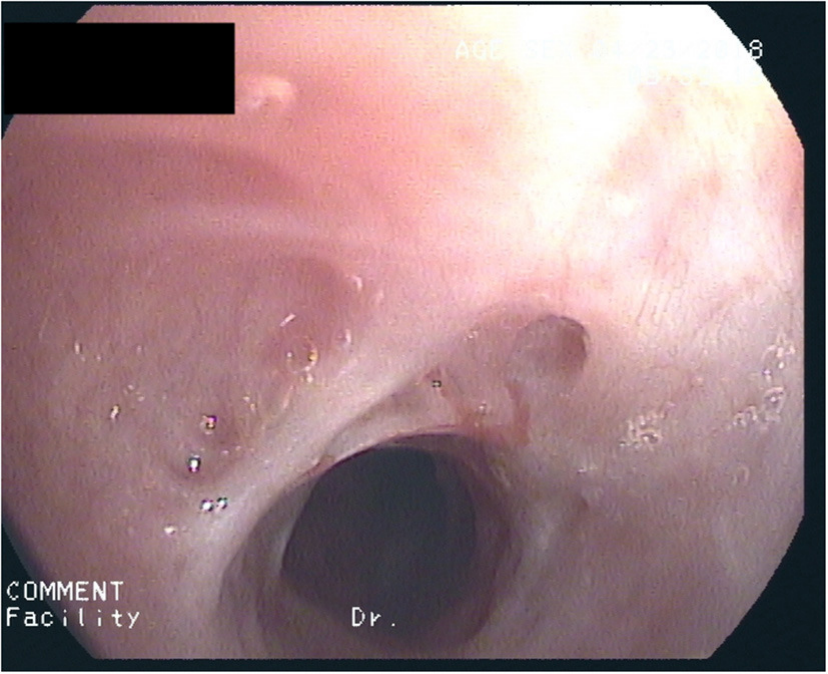
Trachea at start of macrolide-therapy: Thick fibrous strands and marked stenosis.

**Figure 2:**
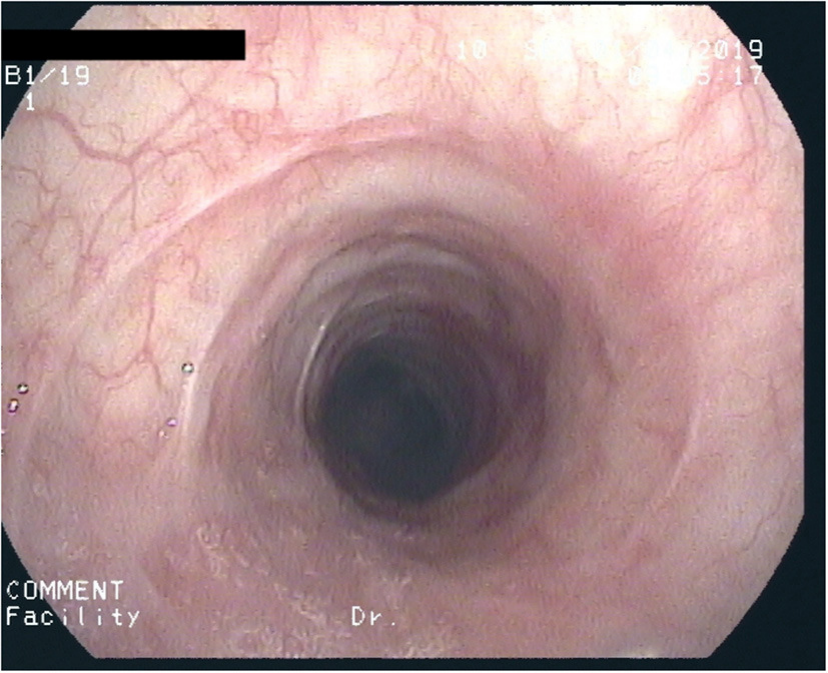
After 1 year of macrolide-therapy: Circular deformed, non-stenotic trachea with considerably diminished scar formation.

After 2½ years of coughing she developed a biphasic resting stridor. The voice remained clear. High-resolution CT of the chest and laboratory testing did not show signs of underlying connective tissue disease (C-ANCA, ANA, lupus anti-ds-DNA, thyroid-antibodies: negative) or Type-1/-3 Allergy (skin-prick-test, sIgE's, precipitating antibodies, BAL-cytology: negative). There were no signs of a chronic inflammatory disease (normal values for: CRP, ESR, Ferritin, Fe, Hb, MCV, amyloid). Spirometry now showed marked inspiratory and expiratory flow limitation. Bronchoscopy revealed progressive scars, constriction of the trachea (Figure 1) as well as chronic mucosal changes of both main and lingula bronchi, with stromal inflammation and epithelial metaplasia, establishing the diagnosis of iSGS. A therapeutic trial with 500 mg p.o. azithromycin (AZT) every day for 1 week, followed by 500 mg every other day was initiated with consent of the parents and the patient. After 2 weeks symptoms diminished considerably, spirometry improved 4 weeks later. Normal spirometry was achieved after 5 months. The patient complained about increasing frontal headache and arthralgia. AZT was reduced to 500 mg 2x/week. After 8 months of therapy, tracheal stenosis had resolved but helically arranged scars were visible. Tracheal mucosa showed ongoing stromal fibrosis and metaplasia with mild chronic inflammation; mucosa of the main and lingula bronchi returned to normal. After discontinuing AZT for 2 weeks without diminishing headache but increasing cough, AZT was started again with 500 mg 2x/week. After 12 months of treatment, histology and endoscopy of the tracheal mucosa showed an increased inflammatory pattern with a normal tracheal diameter (Figure 2). AZT was increased to 3 × 500 mg/week and pantoprazole was added for 4 weeks, again without any positive effect on coughing. AZT was exchanged for roxithromycin 300 mg every other day, because of the probable better anti-inflammatory properties with a lesser dose given. Headache and arthralgia ceased and coughing improved. Laboratory testing showed no signs of inflammation; only discrete blood eosinophilia was noted. After 5 months, roxithromycin was raised to 300 mg/d because of increased coughing. This led to marked and lasting improvement of symptoms. Since macrolide therapy was instituted 3 years ago, spirometry remains normal (Table 1).

**Table 1:** Summary of applied therapies in our patient.

**Time since begin of symptoms**	**Therapy**	**Indication**	**Duration**	**Response to treatment**
7 months	Erythromycin	purulent cough	ND	moderate
	Inhal. steroid	probable asthma	ND	none
	PPI	probable GERD	ND	none
	codeine	cough	4 weeks	poor
8 months	ACA	cough	14 days	none
	Inhal. steroids	cough	ongoing	none
	Next 24 months lost to follow-up			
24 months	Inhal. steroid	cough	ongoing	none
30 months	Diagnosis of iSGS established			
	Azithromycine	Antiinflammation	
	500 mg/d	Cough	1 week	Excellent
	500 mg BID	Cough	4 months	Good
	500 mg 2x/week	Headache and arthralgia	4 weeks	None
	STOP	Headache and arthralgia	2 weeks	None
	500 mg 2x/week	Increased cough	2 weeks	Moderate
36 months	Azithromycine 500 mg 3x/week PPI	Increased tracheal inflammation	4 weeks 4 weeks	None None
37 months	Roxithromycin 300 mg bid	Increased cough	2 years ongoing	Excellent

*ACA, Amoxicillin-Clavulanate; ND, no data; PPI, proton-pump-inhibitor*.

## Review

First published 1972 (6), idiopathic subglottic tracheal stenosis (iSGS) is a rare progressive inflammatory fibrosing disease of the subglottic trachea of unknown origin that leads to progressive airway stenosis (2, 7). It is suspected when other causes of tracheal stenosis are excluded such as congenital narrowing of the trachea, sequelae after intubation, granulomatosis with polyangiitis (Wegener), bilateral vocal cord affections, vocal cord dysfunction, gastro-laryngeal reflux disease and especially asthma (2, 4, 7–11). Since spreading into adjacent bronchi can occur (8, 11–13) some authors call this condition idiopathic tracheal stenosis. The differentiation between the two entities is not well-defined. To rule out connective tissue diseases, ANCA should be determined in every suspected case of iSGS (7, 14–16).

The relative frequency of iSGS is roughly 5–15% of all cases of subglottic stenoses (14, 17). Epidemiological studies calculated the incidence of iSGS as 0.2 per 100,000 (95%CI 0.13–0.3) (2, 17, 18). The age range is 20–85 years (2, 14, 19); mean age at diagnosis is about 47–54 years (2, 20–24) with a median of 47–50 years and an interquartile range from 43–58 years (20, 21, 25).

Caucasians represent more than 95% of the observed cases (18, 21, 26, 27). Almost all patients are female (98–100%) (2, 18, 21–23, 28). Familial occurrence is very rare (2, 4, 29, 30). In a recent study of 810 patients, the rate of familial clustering was calculated at 2.5%, and a non-Mendelian inheritance genetic factor was suggested (31). Published cases in children are sporadic. A summary of the characteristics of published pediatric iSGS patients is given in Table 2.

**Table 2:** Clinical characteristics and treatment in pediatric ISGS cases in the literature.

	**Jazbi (32)**	**Bodart (33)**	**Dedo (19)**	**Valdez (34)**	**Beddow (13)**	**Beddow (13)**	**Modgil (35)**	**Watson (36)**	**Sanders (37)**	**Bodini (38)**	**Hoffman (8)**	**Szadkowski (39)**	**Summary**
Gender	M	M	F	F	M	F	F	F	M	F	M	M	**6M/6F**
Age	2,5	9	15	13	11	11	12	8	12	13	11	11	**2,5-15Y** **(P50** **=** **12Y)**
History/ Comorbidity		streptococcal arthritis				hepatitis B, tracheostomy after severe hemorrhagic pneumonia	started with menarche		TE, AT, atopy	autoimmune thyroiditis	mild epigastric pain (probably ibuprofen induced)	GERD	
Cough/ExIndDyspn/OSAS/Stridor/Wheeze/		E/S			S/W	S	C/E/S	C/D/S/W	E/S/O?	S/O	S	D/S/W	**3 ND 2 C, 4 (E)D, 2O 8S 2W**
Diagnosis established after		12 mo			6 y?		2 y	8 mo	18 mo	3 y	2 mo	4 w	**1mo–6y** **P50** **=** **12mo** **MA 22 mo**
Stenosis diameter			12.25 mm^2^	72%		lumen 2 mm	75% lumen 7 × 4 mm	50% 4.8 mm	Myer-Cotton 3°, pinhole		90%	95% Grade 3	**75–95%**
Stenosis length,-local.		upper trachea, cricoid	2 cm	7 mm	1/2 length of trachea 2,5 cm	distal trachea	2 cm	2 cm			subcricoidal 14 mm		**7–25 mm**
Bronchi involved						+			+				**2/12**
Histology					ulceration	ulceration large lymphoid aggregates							**4/12 ND**
Inflammation		+		+	+	+		+	+		+	+	**8**
Epithelial metaplasia		+			+	+							**3**
Eosinophils/Mastcells					E/M	E/M			E				**3**
Granulation-tissue		+			+	+							**3**
Treatment													
Surgery	+	+			+	+	+						**5**
Dilatation	+			+		+		+	+		+	+	**7**
Laser		+					+						**2**
Inhal Steroid		+					+				+	+	**4 i**
Local Steroids	+								+		+	+	**4 L**
Syst. Steroids		+						+	+	+	+	+	**6 S**
Misc. Therapies	stenting						MIT-C, LABA	amoclav	amoclav, macrolides, AR SABA, adr. inhal.	MTX	AR	AR, H2-blocker	**3 AR 2 amoclav**
# of procedures	2	5	17	1	1	2	1	8	3	1	4	2	
# of relapses		5				1	3	3	2				**7 ND 1–5**
Follow up		24 mo	111 mo	38 mo	10 mo		9 mo		8 mo	1 mo			**0–111 mo** **P50** **=** **10 mo**

The mean time interval between onset of symptoms and diagnosis is 2–3 years (2, 4, 8, 17, 18, 23). Most common symptoms are dyspnea (2, 10, 18) both in rest and during exertion (8, 23, 40, 41), cough, stridor (18, 42), hoarseness, and wheezing (8, 10, 18, 43, 44). Before establishing diagnosis, patients commonly receive an erroneous diagnosis of chronic obstructive pulmonary disease or asthma (10, 24, 45).

A significant number of cases have associated conditions such as hypertension and diabetes mellitus (2, 21). Thyroid disease such as e.g., Hashimoto thyroiditis, Grave's disease, and especially hypothyroidism was also reported in many affected individuals (18, 38, 46).

Flow-Volume loop spirometry typically reveals a box-shaped pattern. The Empey Index (FEV1(ml) to PEFR(l/min) ratio) (47) can be used as a quick indicator of the presence of a tracheal stenosis, with normal values being >7.3–10 (48, 49). Markers, however, show great overlap and variability and do not correlate well-with anatomical grading and the severity of the subglottic stenosis (48, 50–52). Changes in spirometry occur later in the course of the disease when tracheal stenosis exceeds 80% (19, 53).

Flexible and rigid bronchoscopy is the preferred diagnostic tool to demonstrate the extent of tracheo-bronchial involvement, especially the degree of stenosis and macroscopic changes in the trachea. It also allows acquisition of biopsy samples from affected and unaffected areas of the mucosa. The degree of stenosis is usually measured by the Myer-Cotton gradation, which is primarily used for mature, firm and circumferential lesions (5).

Additional information can be acquired through radiologic examinations (44). Plain radiography will typically show the “steeple sign” or an irregular and eccentric stenotic segment. Spiral-CT scans (7, 54) are especially useful for follow-up (55). In children diagnostic radiologic imaging is preferably done by MRI (56). Imaging will usually be considered after endoscopic airway examination.

Histologic evaluation of tracheal tissue biopsies usually shows unspecific acute and chronic active inflammation with epithelial ulceration (5, 13) and subsequent squamous metaplasia (5, 8–10, 13, 44, 57, 58), scattered eosinophilic infiltration (13, 57), normal cartilage (23, 57), and dilatation of mucus glands and ducts (5, 23). Taking biopsies serves mainly to exclude other inflammatory causes of tracheal stenosis (4) as histopathological findings are not pathognomonic in iSGS.

The etiology of iSGS remains largely unclear. GERD is frequently discussed as a leading cause or aggravating factor of iSGS. Reported reflux frequencies range from 11–22% (2, 59) up to 50–71% (59–61). Furthermore, pepsin has been detected in the larynx and trachea in 59% of patients (1). Most GERD patients had no subjective symptoms (3). After surgical tracheal correction no GERD patient required anti-reflux surgery (18). One report shows that anti-reflux therapy greatly lengthened the interval between recurrent interventions (7). Among the few reports in children, one affected boy had an episode of gastritis, probably GERD, prior to presentation (8). A direct causal relationship between iSGS and GERD is questioned by some investigators, who found no difference between history or treatment of reflux in iSGS patients compared to those with granulomatosis-associated tracheal stenosis (12).

In surgically treated iSGS patients, the first tracheal ring was found to be inside the cricoidal lumen. It was hypothesized that this “telescoping” leads to mechanical trauma and, via impairment of circulation, to mucosal ischemia, altogether resulting in inflammation of the subglottic region (14). This hypothesis is probably not always applicable, since in some cases iSGS extends beyond the trachea into the main bronchi. Alternatively, one can speculate that iSGS represents more than one pathologic entity.

As females are predominantly affected, hormonal causes are thought to be involved. A significant proportion of pregnant patients complained about worsening of symptoms (15). Elevated estrogen levels are thought to cause mucosal hyperemia and edema with consequent narrowing of the tracheal lumen (17, 18). Progesterone and estrogen-receptors (ER) have been demonstrated on tracheal fibroblasts in patients with iSGS and in normal controls (17). These findings could not be confirmed in other studies, probably due to a low receptor density or a low sensitivity of the applied techniques (14, 19). Two major subclasses of ER (ER-α, ER-ß) have been found in the subglottic epithelium, glands and ducts whose variable expression may be responsible for differences in wound healing between the sexes. Both subclasses of ER showed increased expression in iSGS-patients (20). There seems to be an imbalance between ER-α, ER-ß, and progesterone-receptors (20, 25). Multiple isoforms of these receptors have been found, but their importance is still not very well-understood.

Pro-inflammatory IL-17 is found in lesions of iSGS-patients. Activation of resident fibroblasts, which differentiate from recruited epithelial cells via a program of epithelial-mesenchymal transition, is promoted through IL-17A and directly drives scar generation. Activated fibroblasts themselves produce cytokines and chemokines which additionally increase the IL-17A driven inflammatory response (26, 27).

There are no predictive parameters regarding the natural history and extension of iSGS (28). It tends to relapse frequently with no increase between the interval of interventions. The length of stenosis (>10 mm) is a major negative prognostic sign (2, 4). Further risks are airway stents, postoperative edema, mitomycin-C application, and vocal cord involvement (18). In one study, the latter developed in 7 of 22 patients (62).

A multimodal therapeutic approach to the iSGS-patient is required. Since etiology and pathophysiology are still unclear, evaluated therapeutic pathways have not yet been established (4). Recommendations are therefore largely based on general treatment options of tracheal stenoses. There is controversy about the initial therapy (10). The success of various therapies is difficult to compare because of differences between the treated populations, the different therapeutic strategies and the pathways followed.

Intervals between successive surgical interventions in a population with mixed causes of subglottic stenosis were neither influenced by different techniques of endoscopic laryngoplasty nor by the severity of the stenosis (40). The long-term success of surgical interventions in patients with a short and mild stenosis was 72.2% (42). In children with acquired subglottic stenosis, primary endoscopic management was successful in 82% of the cases (43). A recent systematic approach noted a relapse rate from 40 to 100% (weighted mean (WM) 68%). The interval between interventions was 2–21 months (WM 12 months) (24). A study of 810 patients with iSGS showed that, compared with other procedures, endoscopic dilatation was associated with higher recurrence rates (21). The most frequent complications are mucosal tearing and bleeding. Severe side effects, such as rupture of airways and blood vessels, have been reported (46).

There are some trials of laser therapy in tracheal stenoses of different etiologies. Laser therapy was almost always applied in combination with other interventions. Most frequently, a CO_2_ laser was used followed by a Nd:YAG laser (2, 24). CO_2_ laser therapy alone had a high recurrence rate (16/50 had more than 10 revision surgeries) and only a moderate success rate (13/50 required tracheotomy) (19). Laser therapy in combination with e.g., mitomycin-C seems to be more effective (63). A trial of pediatric patients with post-intubation stenosis used a holmium laser plus cryotherapy with great success (cure in 15 of 16 children) (64).

Indications for invasive surgical procedures generally are failure of endoscopic approaches (2, 4, 7, 9–11, 61). Several surgical techniques are used, such as single-stage cricotracheal resection according to Grillo (8, 11, 13, 61), cricoplasty with plastic reconstruction of the mucosa, and a combination of resection and enlargement using cartilage grafts from ribs (7, 65). Cricotracheal resection seems to be applicable and safe in children (66, 67), even in infants who weigh <10 kg (2, 68). The preferred definitive treatment is single-stage laryngotracheal resection (14, 17, 69). The published data report success rates between 88 and 97% with relapses in 4–36% of the cases (2, 18, 19, 28, 61, 69, 70). The length of the resection correlated with more frequent relapses and retreatments (2, 21–24, 71). Surgery should be delayed if there are mucosal ulcerations (21, 72). In comparison with endoscopic resection, cricotracheal resection performed better but had the greater perioperative risk and worse voice outcome (18, 21). Rib cartilage usage can stimulate granulation formation, leading to restenosis (2, 18, 19, 21–23). Common postoperative complications apart from restenosis are local granulation, subcutaneous emphysema, wound infections, and pneumonia (18, 29, 30), damage to the recurrent laryngeal nerve, anastomotic dehiscence, and pneumothorax (31, 73). More complications are expected in cases with any type of airway comorbidity (8, 13, 33, 35, 37, 71).

Local corticosteroid injection was associated with significant improvement in the interval between surgeries and had no side effects (2, 8, 17, 18, 23, 74). Similar success rates and side effects were published in a collective with mixed etiologies of subglottic and tracheal stenosis (2, 10, 18, 75). Intralesional injections supressed the hypothalamo-hypophyseal axis for a few days, and systemic side effects with serial injections were not seen (8, 23, 41, 76). Most common side effects were menstrual irregularities, euphoria, and sleep disturbances (18, 77). There is a report of paratracheal phlegmon following a steroid injection (78). Other studies could not demonstrate that surgical intervals were altered by advanced grade of stenosis, dilation technique, or steroid injection (10, 21, 45).

Inhalative steroids are seldom used as adjunct therapy (2, 5, 8, 33, 79). There is doubt about the success of inhalative steroids in the treatment of iSGS, as this disease is regularly mistreated as asthma without success.

Locally applied mitomycin-C is regularly used for its anti-proliferative effects. Several studies showed positive effects such as avoidance of tracheotomy (18, 38, 63) and lengthening of intervention intervals (21, 47, 80). In three studies, however, mitomycin-C was associated with a higher incidence of recurrence, postoperative complications, and no difference in the probability of needing additional operations (2, 18, 71). A recent meta-analysis noted complications, such as subcutaneous and mediastinal emphysema in 9% of the cases. The authors conclude that the use of mitomycin-C is an effective and safe option (81).

Methotrexate (MTX) is used as a key agent in Wegener's granulomatosis and other autoimmune disorders (5, 82). In a small retrospective study MTX improved breathing in 3 of 4 patients, yet symptoms worsened after cessation (83). Low dose MTX was used as adjuvant therapy to surgery in 10 tracheostomy-dependent patients with laryngotracheal stenosis. All participants improved, and three patients could later be decannulated (84). Mild side effects included hair thinning, onychomycosis and shingles.

Rituximab is an established therapy in ANCA-associated vasculitis (55, 82). In combination with other therapies, it was used in 25 iSGS patients. Relapse occurred in seven patients (four within the 1st year) (85).

Besides their antimicrobial effect, macrolides are also known for their anti-inflammatory properties. These have been demonstrated in several pulmonary diseases, such as asthma, bronchiectasis, cystic fibrosis, primary ciliary dyskinesia, community acquired pneumonia and bronchiolitis obliterans (5, 13, 86, 87). Macrolides are also used in skin diseases and inflammatory bowel diseases (5, 8–10, 13, 44, 57, 58, 88). Pro-inflammatory cytokines and mediators are decreased by macrolides, such as TNF-α, IFN-γ, IL-1ß, IL-4, IL-5, IL-6, IL-8. Macrolides diminish neutrophil numbers and concentration of neutrophil elastase. Especially neutrophil-induced inflammation is down-regulated (13, 57, 89). Apoptosis of neutrophils, lymphocytes and eosinophils is enhanced (23, 57, 90–92). One case report of a 12-year old boy with iSGS showed effectiveness of treatment with a macrolide (AZT) (37). In a mouse model, macrolides show different anti-inflammatory characteristics. Whereas, AZT and clarithromycin exhibit only low anti-inflammatory properties, the anti-inflammatory effect of roxithromycin was like that of a NSAID (93). Influencing the local altered microbiome and thus reducing the inflammation could be an additional explanation of the macrolide action (94, 95).

## Summary

Our patient developed iSGS at a very young age. The disease extended into the main and lingula bronchi. She has autoimmune disorders such as chronic spontaneous urticaria and celiac disease. GERD could not be demonstrated nor was repeated anti-reflux therapy successful. Antibiotic therapy with erythromycin in the very early stage of the disease suggested a possible therapeutic effect of macrolides. Based on the observed response to erythromycin and in agreement with the aforementioned studies (37) we started a trial with azithromycin, later substituted by roxithromycin. As symptoms rapidly diminished and spirometry as well as tracheal lumen normalized, other therapeutic options were no longer considered (Table 1). We expect that iSGS in our patient will likely not be cured by macrolide therapy, so our goal is to postpone interventions, such as repeated interventional bronchoscopies and ultimately crico-tracheal resection, at least until adulthood.

## Conclusion

As iSGS is a rare and potentially long-term debilitating disease, prospective multi-center studies have been initiated to determine the best diagnostic and therapeutic approach (2, 59, 96). This case report highlights that the use of macrolides should be considered as a viable treatment option, especially in pediatric patients.

Written consent of the parents and patient was obtained for potential scientific and educational use of the findings and images pertaining to her disease.

## Data availability statement

The raw data supporting the conclusions of this article will be made available by the authors, without undue reservation.

## Ethics statement

Ethical review and approval was not required for the study on human participants in accordance with the local legislation and institutional requirements. Written informed consent to participate in this study was provided by the participants' legal guardian/next of kin. Written informed consent was obtained from the minor(s)' legal guardian/next of kin for the publication of any potentially identifiable images or data included in this article.

## Author contributions

WT wrote the manuscript. HW and JT contributed to literature and manuscript revision. HW and GH approved the submitted version. All authors contributed to manuscript revision, read, and approved the submitted version.

## Conflict of interest

The authors declare that the research was conducted in the absence of any commercial or financial relationships that could be construed as a potential conflict of interest.

## Publisher's note

All claims expressed in this article are solely those of the authors and do not necessarily represent those of their affiliated organizations, or those of the publisher, the editors and the reviewers. Any product that may be evaluated in this article, or claim that may be made by its manufacturer, is not guaranteed or endorsed by the publisher.
